# Patterns and trends of alcohol consumption in rural and urban areas of China: findings from the China Kadoorie Biobank

**DOI:** 10.1186/s12889-019-6502-1

**Published:** 2019-02-20

**Authors:** Pek Kei Im, Iona Y. Millwood, Yu Guo, Huaidong Du, Yiping Chen, Zheng Bian, Yunlong Tan, Zhendong Guo, Shukuan Wu, Yujie Hua, Liming Li, Ling Yang, Zhengming Chen, on behalf of the China Kadoorie Biobank (CKB) collaborative group

**Affiliations:** 1 Clinical Trial Service Unit and Epidemiological Studies Unit (CTSU), Nuffield Department of Population Health, University of Oxford, Oxford, UK; 2 Medical Research Council Population Health Research Unit (MRC PHRU), Nuffield Department of Population Health, University of Oxford, Oxford, UK; 3 Chinese Academy of Medical Sciences, Beijing, China; 4 Meilan CDC, Haikou, Hainan China; 5 Suzhou CDC, Suzhou, Jiangsu China; 6 Department of Epidemiology and Biostatistics, School of Public Health, Peking University, Beijing, China

**Keywords:** Alcohol, China, Patterns, Trends

## Abstract

**Background:**

In China, alcohol consumption has increased significantly in recent decades. Little evidence exists, however, about temporal trends in levels and patterns of alcohol consumption and associated factors in adult populations.

**Methods:**

In 2004–08, the China Kadoorie Biobank recruited ~ 512,000 adults (41% men, mean age 52 years [SD 10.7]) from 10 (5 urban, 5 rural) geographically diverse regions across China, with ~ 25,000 randomly selected participants resurveyed in 2013–14. The self-reported prevalence and patterns (e.g., amount, beverage type, heavy drinking episodes) of alcohol drinking at baseline and resurvey were compared and related to socio-demographic, health and other factors.

**Results:**

At baseline, 33% of men drank alcohol at least weekly (i.e., current regular), compared to only 2% of women. In men, current regular drinking was more common in urban (38%) than in rural (29%) areas at baseline. Among men, the proportion of current regular drinkers slightly decreased at resurvey (33% baseline vs. 29% resurvey), while the proportion of ex-regular drinkers slightly increased (4% vs. 6%), particularly among older men, with more than half of ex-regular drinkers stopping for health reasons. Among current regular drinkers, the proportion engaging in heavy episodic drinking (i.e., > 60 g/session) increased (30% baseline vs. 35% resurvey) in both rural (29% vs. 33%) and urban (31% vs. 36%) areas, particularly among younger men born in the 1970s (41% vs. 47%). Alcohol intake involved primarily spirits, at both baseline and resurvey. Those engaging in heavy drinking episodes tended to have multiple other health-related risk factors (e.g., regular smoking, low fruit intake, low physical activity and hypertension).

**Conclusions:**

Among Chinese men, the proportion of drinkers engaging in harmful drinking behaviours increased in the past decade, particularly among younger men. Harmful drinking patterns tended to cluster with other unhealthy lifestyles and health-related risk factors.

**Electronic supplementary material:**

The online version of this article (10.1186/s12889-019-6502-1) contains supplementary material, which is available to authorized users.

## Background

Globally, alcohol is the seventh leading risk factor for poor health, accounting for 4.2% of total disability-adjusted life years and 5.2% of deaths in 2016 [1]. In China, the prevalence of alcohol drinking is lower than in many Western populations, particularly among women [2], which may reflect in part differences in cultural attitudes toward alcohol. While alcohol is traditionally used in festivals and celebration in China, it is also commonly used in the Chinese business world to maintain good relations, particularly by men [3]. Also, social drinking is traditionally widely acceptable among men but not among women in China [3–5]. Moreover, genetic factors may also play a role, as the unpleasant flushing response upon alcohol drinking due to a deficiency in metabolizing alcohol is common in Chinese populations [6]. Despite this, there have been dramatic increases in alcohol availability, production and per capita consumption in China over recent decades due to rapid economic development and urbanisation [3, 7, 8], which may lead to an increased burden of chronic diseases such as alcohol-related cancers and chronic liver disease, as well as accidents and injuries. Questions remain, however, about changes in patterns of alcohol drinking (e.g., drinking frequency, heavy episodic drinking, beverage types) in adult Chinese populations. Most published studies on alcohol drinking in Chinese populations tended to have small sample sizes, were conducted before 2010, or lacked detailed investigation of drinking patterns [9–17]. Moreover, heterogeneity in the geographical regions covered and definitions used makes it difficult to compare changes in alcohol consumption patterns across previous studies.

While several cross-sectional studies in China reported on drinking patterns during 1994–2007 [9, 12–15], and on drinking prevalence in 2008 and 2011 [10, 16], only one study has attempted to explore the trends of alcohol drinking in general population by using five survey waves from the China Health and Nutrition Survey (CHNS) between 1993 and 2006 [17]. A recently published study explored the increasing trends of alcohol use from 2000 to 2014 using the Chinese Longitudinal Healthy Longevity Survey (CLHLS), but only focused on older adults aged 60 years or above in China [18]. Since 2007, there have been no reported nationwide studies on drinking patterns and trends in the general population in China. The CHNS study, with around 9000 participants from nine provinces in each wave, showed that levels of alcohol intake and daily drinking in men increased during 1997–2000 but then remained steady until 2006 [17]. Yet, the World Health Organisation (WHO) global status report on alcohol and health, based on recorded alcohol production and trade data, demonstrated a striking increase in per capita alcohol consumption in China between 1978 and 2010 [8, 19]. Large nation-wide epidemiological surveys are needed to provide new evidence on the recent trends of drinking prevalence, and consumption level and drinking patterns among regular drinkers in China.

Longitudinal studies conducted in Western populations have shown that alcohol drinking varies with age throughout the life-course [20–22]. However, to our knowledge, there is no study investigating changes in drinking behaviour at the individual level in China. Furthermore, as stopping drinking can reduce the risk of alcohol-related harm [23], knowledge about the motivations for alcohol cessation could help to inform the development of effective alcohol drinking interventions. However, evidence on the factors and reasons for stopping alcohol drinking in China is limited.

Using data from the China Kadoorie Biobank (CKB) prospective cohort study, we aim to assess: 1) the temporal trends in the prevalence and patterns of alcohol drinking between the baseline survey in 2004–8 and a resurvey in 2013–14, and 2) the socio-demographic, health and other factors associated with changes in drinking prevalence and patterns over this time period.

## Methods

### Study design

The China Kadoorie Biobank (CKB) is a nationwide prospective blood-based cohort study involving 0.5 million participants recruited from 10 geographically defined regions across China, established to investigate genetic and non-genetic causes of many common chronic diseases in the Chinese population. Details of the CKB study design and survey methods have been described previously [24, 25]. Briefly, the baseline survey was conducted in 2004–8 among 512,891 men and women aged 30–79 years recruited from five rural and five urban regions across China (response rate~ 30%), which were selected to cover a wide range of risk factor exposures and disease patterns. The baseline survey involved an interviewer-administered questionnaire, collecting information on demographic and socioeconomic status, lifestyle behaviours (alcohol drinking, smoking, diet, physical activity) and medical history, physical measurements and collection of blood samples. Five-year periodic resurveys of (~ 5%) randomly selected surviving participants were conducted after the baseline survey using similar procedures. For the analyses of temporal trends, we used data from the baseline survey in 2004–8 of 512,891 participants and the resurvey in 2013–14 (mean 8 years [SD 0.8] after baseline) which enrolled 24,996 participants. Among those potentially eligible for the 2013–14 resurvey, the response rate was ~ 75% and 0.7% of participants had died before the time of resurvey. Ethical approval for the study was obtained from the ethics committees of the University of Oxford and the Chinese Centre for Disease Control and Prevention. All participants provided written informed consent at baseline and resurveys.

### Assessment of alcohol drinking

Detailed information on questionnaire assessment of alcohol consumption in the CKB study has been described previously [26]. In brief, based on the frequency of alcohol drinking during the past year and prior to the past year, participants were classified into five main drinking categories (see Additional file 1: Table S1 for detailed classifications): abstainers; ex-weekly drinkers; reduced-intake drinkers; occasional drinkers; and current weekly drinkers. Current weekly drinkers were asked further questions about their drinking patterns including: (1) drinking frequency; (2) beverage types; (3) amount consumed for each type on a typical drinking day, on special occasions, and the last time they drank; (4) experience of problem drinking indicators; (5) time of drinking in relation to meals; (6) experience of flushing response after drinking; and (7) age of starting drinking weekly. At the resurvey only, ex-weekly and reduced-intake drinkers were asked about their reasons for stopping or reducing drinking.

The amount of pure alcohol consumed in grams per session was calculated according to the beverage type and amount drunk on the last time they drank alcohol (see Additional file 1: Tables S2 and S3 for information on data quality), based on the assumption of the following alcohol content by volume (*v*/v) typically seen in China [3]: beer 4%, grape wine 12%, rice wine 15%, weak spirits 38% and strong spirits 53%. Consumption on special occasions was calculated in the same way. Heavy episodic drinking was defined as consuming more than 60 g of alcohol on one occasion for men, and more than 40 g for women [27]. The following indicators of problem drinking were reported in the past month: drinking in the morning; unable to work due to drinking; depressed/irritated or loss of control due to drinking; unable to stop drinking; having shakes when stopping drinking.

To describe changes in drinking status at the individual level between baseline and resurvey, six categories were defined: stable non-drinkers; starters; stable drinkers; stoppers; decreased-intake drinkers; and increased-intake drinkers (detailed definition shown in Additional file 1: Table S1).

### Assessment of multiple health-related risk factors

To assess the presence of other health-related risk factors in participants, a risk factor index was derived by summing the individual scores of four major risk factors (1 = yes, 0 = no): regular smoking, lack of daily fresh fruit intake, low physical activity, and hypertension (see Additional file 1: Table S1 for detailed classifications).

### Statistical analyses

As alcohol drinking prevalence and behaviour differed significantly between men and women in CKB, all analyses were conducted separately in men and women. Temporal trends in alcohol drinking were assessed using cross-sectional data from all participants recruited at baseline (*n* = 512,891) and from participants attending the resurvey (*n* = 24,996). The crude prevalence of alcohol drinking and mean consumption were calculated at each survey. Among subgroups defined by socio-demographic factors and the risk factor index, the prevalence of alcohol drinking was directly standardized to the age and region structure of the study population. As drinking patterns were assessed in weekly drinkers, the relevant variables were standardized according to the age and region structure of weekly drinkers. Due to the small number of female weekly drinkers (n_baseline_ = 6248 and n_resurvey_ = 292), analyses of drinking patterns by subgroups were conducted in male weekly drinkers only (n_baseline_ = 69,904 and n_resurvey_ = 2732).

Longitudinal analyses of changes in drinking status were conducted among men who attended both surveys (*n* = 9569). A similar standardization approach was applied as above to obtain the prevalence of each status change category standardised to the age and region structure of the longitudinal subset. The statistical association between factors and change in drinking status was tested using logistic regression among baseline non-drinkers and multinomial logistic regression among baseline drinkers. The reasons for stopping weekly drinking were assessed among previous weekly drinkers (i.e. ex-weekly and reduced-intake drinkers) in the resurvey (*n* = 1166). Crude percentages of each of the reasons reported were calculated overall, and by socio-demographics and health factors. Among men who were current weekly drinkers at both baseline and resurvey, a dependent sample t-test was used to test for the statistical significance of change in alcohol consumption at individual level over time. For a quality check of the self-reported alcohol data, associations of blood pressure with alcohol consumption were assessed using linear regression adjusted for age, study region, education, income, smoking category, physical activity and month of recruitment. Data from the CKB Data Release 10 was used in this study. All analyses were performed in SAS version 9.4.

## Results

Of the 512,891 participants recruited at baseline, the mean age was 52 years (SD 10.7), 41% were men and 56% were from rural areas. The subset of participants resurveyed (*n* = 24,996) was broadly representative of the baseline study population in terms of the distribution of baseline characteristics of sex, birth cohorts, area, education, and health and lifestyle factors (Additional file 1: Table S4). Though the response rates at resurvey were highly consistent across baseline drinking groups, there was slight variation across birth cohorts from 68% in the oldest and youngest cohorts to 79% among the middle cohorts (Additional file 1: Table S5). Between baseline and resurvey, there was an increase in household income in the study population (Table 1).

**Table 1:** Cross-sectional characteristics of participants at baseline (2004–8) and resurvey (2013–14)

Characteristics*	Men		Women	
	Baseline (*N* = 210,259)	Resurvey (*N* = 9569)	Baseline (*N* = 302,632)	Resurvey (*N* = 15,427)
Socio-demographic characteristics				
Mean age, years	52.4	59.7	51.0	58.5
Birth cohorts, %				
< 1940	13.8	11.6	10.3	8.6
1940–1949	22.4	24.2	20.6	22.2
1950–1959	30.9	32.9	32.2	34.4
1960–1969	27.9	27.5	31.3	30.5
≥ 1970	4.9	3.9	5.7	4.3
Area, %				
Rural	56.6	56.8	55.4	57.1
Highest education, %				
No formal education	8.9	9.2	25.3	27.1
Primary school	33.4	33.4	31.4	31.4
Middle or high school	49.9	49.2	38.8	37.4
Technical school/college or above	7.9	8.2	4.5	4.2
Household income (yuan/year), %				
< 10,000	26.0	8.2	29.8	9.0
10,000-19,999	28.3	11.2	29.6	13.0
20,000-34,999	25.4	17.9	24.2	20.2
35,000+	20.2	62.7	16.5	57.8
Health and lifestyle factors				
Regular smoking, %	61.1	51.0	2.4	1.6
Daily fruit intake, %	23.0	39.0	31.8	48.8
Physical activity, mean MET hours/day	22.0	21.1	20.4	18.4
Self-reported good health^a^, %	49.2	47.0	43.3	41.8
Prior disease^b^, %	22.6	31.7	22.1	33.9
Satisfied with life^c^, %	69.7	82.4	67.7	80.5
Physical measurements				
Mean SBP, mmHg	132.8	136.9	129.9	136.3
Mean DBP, mmHg	79.2	79.6	76.8	77.5
Mean heart rate, beats/minute	77.7	76.6	79.7	78.2
Mean BMI, kg/m^2^	23.4	24.0	23.8	24.3
Mean WHR	0.9	0.9	0.9	0.9
Mean standing height, cm	165.2	164.7	154.1	153.5

*BMI* body mass index, *S/DBP* systolic/diastolic blood pressure, *MET* metabolic equivalent task, *WHR* waist-hip ratio

*Characteristics were based on the characteristics of the participants collected at each of the baseline and the resurvey

^a^Reporting good or excellent self-rated health status

^b^Diagnosed with one or more of: coronary heart disease, stroke, transient ischaemic attack, diabetes, cancer, tuberculosis, chronic hepatitis/cirrhosis, rheumatoid arthritis, peptic ulcer, chronic respiratory disease, gallstone/gallbladder disease, kidney disease

^c^Reporting being satisfied or very satisfied with life

At baseline, 33% of men but only 2% of women reported drinking alcohol at least weekly (i.e., current regular drinkers), whereas a third of men and women drank occasionally (Table 2). Among male weekly drinkers, mean alcohol intake was 50 g/session (based on the last drinking day), with 62% drinking daily or almost daily (6-7 days/week). The majority of male weekly drinkers engaged in heavy episodic drinking on special occasions (84%), with 30% doing so on the last drinking day, and 24% reported at least one indicator of problem drinking (e.g., drinking in the morning, unable to work due to drinking etc). Female weekly drinkers had lower mean consumption and were less likely to report heavy episodic drinking or problems related to their drinking. Increased alcohol consumption was associated with higher blood pressure at each survey (Additional file 1: Table S3). In both sexes, strong spirits were the most common beverage type consumed. Most weekly drinkers usually drank with meals (86% overall, in men and women), which was broadly consistent across study areas, except for one rural region where ~ 20% drank with meals (Additional file 1: Table S6).

**Table 2:** Prevalence and patterns of alcohol consumption at baseline (2004–2008) and resurvey (2013–2014), by sex

	Men		Women	
	Baseline	Resurvey	Baseline	Resurvey
Overall				
Number of participants	210,259	9569	302,632	15,427
Drinking categories, %				
Abstainer	20.4	34.9	63.6	82.0
Ex-weekly	3.8	6.3	0.4	0.7
Reduced-intake	4.9	3.8	0.4	0.5
Occasional	37.7	26.4	33.5	14.9
Current weekly	33.2	28.6	2.1	1.9
Among current weekly drinkers				
Number of participants	69,904	2732	6248	292
Types consumed^^^, %				
Strong spirit (≥40% alcohol) only	41.5	41.8	47.6	39.4
Weak spirit (< 40% alcohol) only	19.8	16.0	11.4	12.0
Beer only	20.1	13.5	22.6	12.3
Rice wine or grape wine only	11.0	12.3	15.7	27.1
Mixed	7.7	16.3	2.7	9.2
Mean consumption, g/session^^^	49.9	55.6	22.7	24.1
Number of drinking days per week, %				
1–2	19.9	14.9	33.1	21.9
3–5	18.0	14.0	21.6	15.4
6–7	62.1	71.1	45.2	62.7
Types consumed on special occasions, %				
Strong spirit (≥40% alcohol) only	35.7	35.8	41.4	33.9
Weak spirit (< 40% alcohol) only	16.3	14.7	9.9	10.6
Beer only	10.9	8.4	16.7	11.0
Rice wine or grape wine only	6.6	9.3	12.3	25.7
Mixed	30.5	31.8	19.6	18.8
Mean consumption on special occasions, g/occasion	147.4	116.3	55.1	41.3
Drinking patterns, %				
Drinking outside meal.	14.1	17.6	13.8	20.9
Heavy episodic drinking^^a^	29.8	34.7	18.2	17.8
Heavy episodic drinking on special occasions^a^	83.6	72.9	57.1	43.8
Problem drinking indicator(s)^b^	23.9	23.8	9.8	9.2
Flushing response after drinking	17.9	15.5	23.6	13.7
Mean age started weekly drinking	28.7	29.3	37.7	40.4

^a^Heavy drinking episode is defined as drinking > 60 g of pure alcohol in one session for men and > 40 g for women

^b^Reporting one or more in the past month of: drinking in the morning; unable to work or do anything due to drinking; depressed, irritated or lost control due to drinking; couldn’t stop drinking; had shakes when stopped drinking

^^^Based on alcohol intake data reported on the last time the participants drank

Among men, the prevalence of weekly drinking was lower at resurvey than at baseline (33% baseline vs. 29% resurvey), as was occasional drinking (38% vs. 26%) (Table 2). Similar differences were also evident in women for occasional drinking (34% vs. 15%). Among male weekly drinkers, however, there were increases in mean consumption on the last drinking day (50 g/session vs. 56 g/session), and in the prevalence of heavy episodic drinking (30% vs. 35%) and daily drinking (62% vs. 71%) during the same period. Further analyses among men who drank weekly at both baseline and resurvey showed that mean consumption increased by 3.7 g/session (*p* = 0.004) (Additional file 1: Table S7). While the most common beverage type was strong spirits only (consumed by ~ 42% of male weekly drinkers at both surveys), there was an increase in the proportion of drinkers consuming multiple beverage types (8% vs. 16%), who tended to have a higher intake per session than those drinking a single beverage type (Additional file 1: Table S8). Similar drinking patterns and temporal trends were observed in the subset of participants who were involved in both the baseline survey and resurvey (Additional file 1: Table S9).

**Fig. 1:**
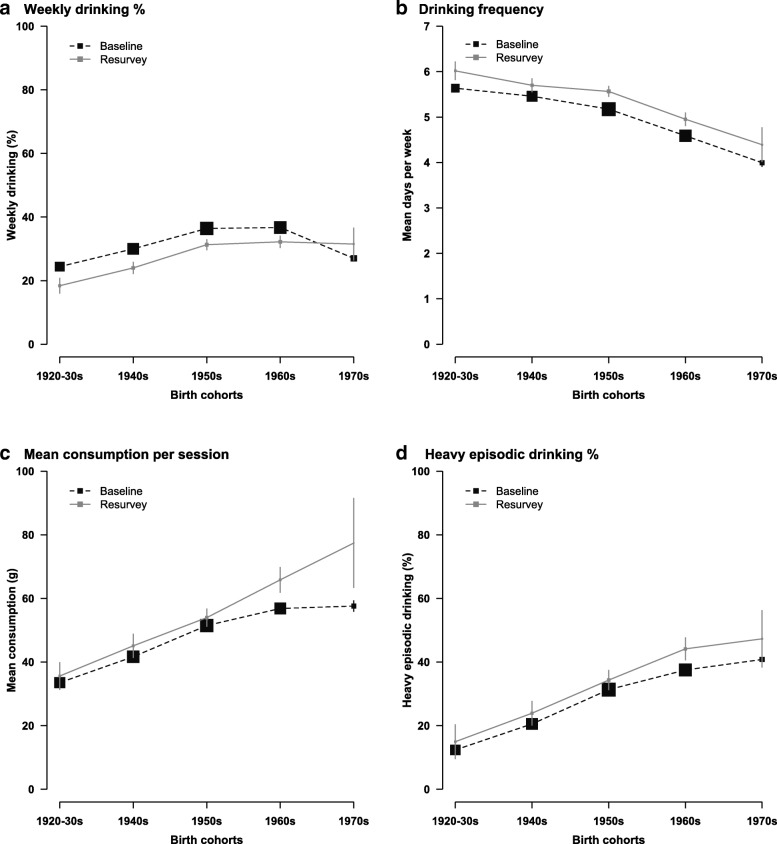
Alcohol drinking characteristics in male weekly drinkers in 2004–8 and 2013–4, by birth cohort. Prevalence and mean were adjusted for regions. Size of boxes is proportional to the sample size of the respective birth cohort. Error bars are 95% confidence intervals. Mean consumption per session (g/session) and heavy episodic drinking was based on alcohol intake data reported on the last time the participants drank. Heavy episodic drinking is defined as drinking > 60 g of pure alcohol in one session for men. All men at baseline (*n* = 210,259) and resurvey (*n* = 9569) were included in (**a**). All male weekly drinkers at baseline (*n* = 69,904) and resurvey (*n* = 2732) were included in (**b**-**d**)

Among men, the patterns and trends of alcohol drinking differed by birth cohort (Fig. 1a-1d). At baseline, weekly drinking prevalence was highest in men born in the 1950s–60s (36%) (Fig. 1a). Over the study period, the prevalence of weekly drinking decreased in most birth cohorts, except for the youngest cohort born in the 1970s who had a slightly higher prevalence of weekly drinking at resurvey (27% vs. 32%), which may be due to the effects of age (Additional file 1: Figure S1). Among male weekly drinkers in both surveys, drinking frequency was higher among older birth cohorts (Fig. 1b), but mean consumption per session and the prevalence of heavy episodic drinking were higher among younger birth cohorts and increased over time in these groups (58 g/session vs. 77 g/session and 41% vs. 47% respectively in men born in the 1970s) (Fig. 1c, d).

Drinking patterns also differed by study area and socio-economic status in men at both surveys. The prevalence of weekly drinking was higher in urban areas, while the proportion of daily drinkers and men reporting problem drinking indicators among those who drank at least weekly was higher in rural areas and lower socio-economic groups (Additional file 1: Tables S10 and S11). Over time, a more marked increase in mean alcohol consumption was observed among urban drinkers. The prevalence of men who reported at least one problem drinking indicator increased in urban areas and particularly in younger urban drinkers, but generally declined in rural areas (Additional file 1: Table S12). Furthermore, younger generations also had a higher tendency to drink beer or multiple beverage types, with a notable rise in the proportion of mixed-beverage drinkers between baseline and resurvey (10% vs. 25% in those born in the 1970s) (Additional file 1: Figure S2).

**Fig. 2:**
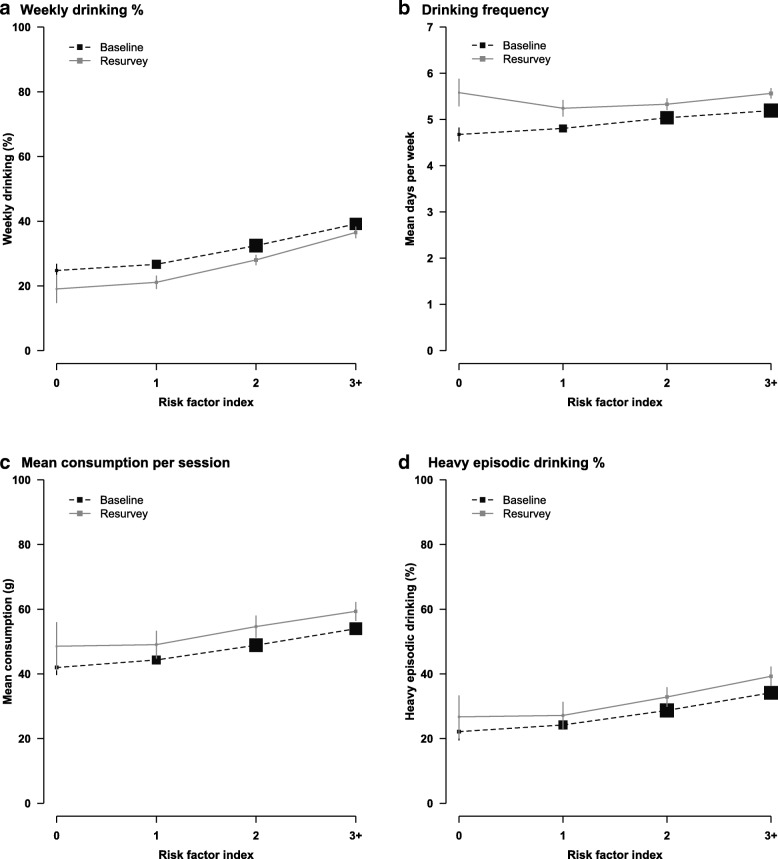
Alcohol drinking characteristics in male weekly drinkers in 2004–8 and 2013–4, by health-related risk factor index. Prevalence and mean were adjusted for age and regions. Size of boxes is proportional to the sample size of the respective risk factor index group. Error bars are 95% confidence intervals. Mean consumption per session (g/session) and heavy episodic drinking was based on alcohol intake data reported on the last time the participants drank. Heavy episodic drinking is defined as drinking > 60 g of pure alcohol in one session for men. Risk factor index was derived by summing the individual scores of each of the four risk factors (0 if no, 1 if yes): regular smoking, lack of daily fruit intake, hypertension, low physical activity. All men at baseline (*n* = 210,259) and resurvey (*n* = 9569) were included in (**a**). All male weekly drinkers at baseline (*n* = 69,904) and resurvey (*n* = 2732) were included in (**b**-**d**)

Having multiple health-related risk factors (i.e. regular smoking, low physical activity, low fresh fruit intake, hypertension) was correlated with alcohol drinking, with weekly drinking prevalence, drinking frequency, mean consumption and heavy episodic drinking prevalence all increasing with the number of risk factors in men at both surveys (Fig. 2a-d). When hypertension was removed from the risk factor index, the correlation persisted between unhealthy lifestyles and alcohol drinking (Additional file 1: Figure S3).

Among 9569 men who attended both surveys, during 2004–8 and 2013–14, 40% of them continually drank and 18% did not drank alcohol on both occasions, 23% stopped drinking and 4% started drinking since baseline (Table 3). Men who were older, living in rural areas or with poor self-reported health status at resurvey were more likely to remain non-drinking as well as to have stopped drinking. The results were consistent though less apparent when analyses used baseline rather than resurvey characteristics (Additional file 1: Table S13), suggesting that changes in health status and ageing over the study period was correlated with stopping drinking. Similarly, among the 1166 ex-weekly or reduced-intake drinkers in the resurvey, over half of them reported existing physical illness as their main reason for stopping weekly drinking, particularly in those who were older or had prior diseases (Table 4). Other reported reasons for stopping drinking were money (24%), future health concerns (6%), and other reasons not related to financial or health concerns (19%).

**Table 3:** Changes in drinking status by socio-demographic characteristics and health factors among men from baseline to resurvey

Characteristics*	N	Non-drinkers (abstainers, ex-weekly drinkers) at baseline^^^		Drinkers (reduced-intake, occasional and weekly drinkers) at baseline^#^			
		Stable non-drinker %	Starter %	Stable drinker %	Stopper %	Decreased-intake drinker %	Increased-intake drinker %
All men	9569	18.3	4.2	40.0	22.9	8.2	6.3
Socio-demographic characteristics							
Birth cohorts							
< 1940	1106	33.0	4.3	24.0	30.6	3.9	4.3
1940–1949	2311	23.1	4.3	32.4	28.3	6.9	5.2
1950–1959	3149	16.3	3.8	43.7	22.1	8.2	6.0
1960–1969	2632	11.3	4.6	47.3	18.1	10.7	8.0
≥ 1970	371	8.3	3.7	50.3	16.0	11.7	10.0
Area							
Rural	5433	21.9	4.3	36.3	24.5	6.9	6.1
Urban	4136	13.6	4.1	44.8	20.9	10.0	6.6
Highest education							
Primary or below	4076	20.1	4.2	36.9	24.1	8.6	6.1
Middle or above	5493	16.7	3.9	42.1	22.5	8.4	6.4
Household income (yuan/year)							
< 35,000	3573	21.5	3.9	37.0	23.9	6.8	7.0
35,000+	5996	16.8	4.4	40.8	22.9	8.9	6.2
Health factors							
Self-reported health status^a^							
Good	4498	16.7	4.4	43.2	20.0	8.4	7.3
Poor	5071	19.9	4.0	37.0	25.6	8.1	5.4
Prior disease^b^							
No	6533	17.0	3.9	42.6	21.3	8.5	6.7
Yes	3036	21.4	5.1	34.3	26.4	7.6	5.1
Risk factor index score^c^							
0	469	18.1	5.4	36.7	25.5	7.9	6.3
1	1962	18.3	4.6	36.8	24.9	9.6	5.8
2	3559	18.4	4.7	40.1	23.0	7.4	6.4
3+	3579	17.4	3.5	43.1	21.1	8.2	6.7

Prevalence at subgroup levels is adjusted for age and regions as appropriate

*Except for age and regions, characteristics were based on the characteristics of the participants collected at the resurvey

^^^Among baseline non-drinkers, associations between change in drinking status and factors were tested by logistic regression adjusting for age and region: *p* < 0.001 for trend across birth cohorts and *p* < 0.02 for heterogeneity across regions, income and self-reported health

^#^Among baseline drinkers, associations between change in drinking status and factors were tested by multinomial logistic regression adjusting for age and region: *p* < 0.02 across all variables except education

^a^Poor self-reported health status include those who reported fair or poor self-rated health; Good self-reported health status include those who reported good or excellent self-rated health

^b^Diagnosed with one or more of: coronary heart disease, stroke, transient ischaemic attack, diabetes, cancer, tuberculosis, chronic hepatitis/cirrhosis, rheumatoid arthritis, peptic ulcer, chronic respiratory disease, gallstone/gallbladder disease, kidney disease

^c^Derived by summing the individual scores of each of the four risk factors (0 if no, 1 if yes): regular smoking, lack of daily fruit intake, hypertension, low physical activity

**Table 4:** Reasons for stopping drinking by socio-demographic characteristics and health factors among previous weekly drinkers at resurvey (2013–2014)

Characteristics*	N	Reasons			
		Existing illness (%)	Money (%)	Future health^^^ (%)	Other^#^ (%)
Overall	1166	51.9	23.6	5.8	18.7
Socio-demographic characteristics					
Gender					
Men	970	53.1	24.3	6.3	16.3
Women	196	45.9	19.9	3.6	30.6
Birth cohorts					
< 1940	174	56.9	17.8	5.7	19.5
1940–1949	408	57.4	21.1	5.4	16.2
1950–1959	381	47.2	27.3	7.1	18.4
≥ 1960	203	45.3	26.6	4.4	23.6
Area					
Rural	687	60.3	21.5	6.3	11.9
Urban	479	39.9	26.5	5.2	28.4
Highest education					
Primary or below	589	60.1	20.4	7.0	12.6
Middle or above	577	43.5	26.9	4.7	25.0
Household income (yuan/year)					
< 35,000	487	58.5	23.2	5.3	12.9
35,000+	679	47.1	23.9	6.2	22.8
Health factors					
Self-reported health status^a^					
Good	408	42.9	26.0	7.1	24.0
Poor	758	56.7	22.3	5.1	15.8
Prior disease^b^					
No	618	44.8	27.0	6.5	21.7
Yes	548	59.9	19.7	5.1	15.3

*Except for age and regions, characteristics were based on the characteristics of the participants collected at the resurvey

^^^Included health concerns about future illness and doctor’s advice

^#^Included family disapproval and other reasons apart from health and financial concern

^a^Poor self-reported health status include those who reported fair or poor self-rated health; Good self-reported health status include those who reported good or excellent self-rated health

^b^Diagnosed with one or more of: coronary heart disease, stroke, transient ischaemic attack, diabetes, cancer, tuberculosis, chronic hepatitis/cirrhosis, rheumatoid arthritis, peptic ulcer, chronic respiratory disease, gallstone/gallbladder disease, kidney disease

## Discussion

This large study examines recent patterns and trends of alcohol drinking and factors associated with stopping drinking in urban and rural areas of China. Overall, there was a modest decline in weekly drinking prevalence between 2004 and 2013 as the study population became older, but a modest increase in mean consumption, drinking frequency and heavy episodic drinking prevalence among men who drank alcohol weekly. Younger age and having multiple health-related risk factors were important correlates of heavy drinking patterns. Older and less healthy men were more likely to stop drinking, suggesting that existing illness was a major reason for stopping drinking in this Chinese population.

The findings on drinking prevalence in our study are broadly consistent with previous nationwide cross-sectional surveys at particular time point in China during 2002–2011 [10, 12, 13]. A study involving 160,000 participants aged 15+ years from the China National Nutrition and Health Survey (CNNHS) found a similar weekly drinking prevalence of 39.6% in men and 4.5% in women in 2002 [13]. The latest wave of the CHNS in 2011 (*n* = 12,658) showed a prevalence of past-year ever drinking of 59% in men, which is comparable to that in our resurvey, but did not report on drinking frequency or other drinking patterns [10]. With detailed information on drinking patterns at two surveys approximately 8 years apart, our study showed a modest decrease in weekly drinking prevalence over time since 2004, similar to findings observed in a previous study of US men and women aged 45–64 years, sampled 6 years apart [20]. This may be due to ageing in the study population and the “sick-quitter” effect [28], as suggested by the associations of older age and poor health with stopping drinking in our study. Interestingly, despite the decreased drinking prevalence, we observed modest increases in mean alcohol consumption per drinking session and in the prevalence of daily drinking and heavy episodic drinking, among male weekly drinkers, which is consistent with the increasing per capita alcohol consumption in China shown in the WHO report [8, 19]. This is in contrast to patterns observed in many Western populations, for example, a decreasing trend in alcohol consumption levels during 1990s–2010s among adults aged 18–85 years in Switzerland [29] and in other southern, central-western and western European countries [30], and a reduction in frequent drinking (5+ days in the previous week) among adults aged over 16 years during 2005–2017 in the United Kingdom [31].

Previous studies in China conducted in the 2000s reported that harmful drinking patterns peaked between the ages of 30–60 years in men [12, 15]. These studies, however, used rather low thresholds to define harmful drinking (e.g., >25 g or >50 g/day for excessive or binge drinking in men). In our study, using the WHO recommended threshold for high-risk drinking episodes (>60 g/session in men) [27], we showed that younger men were more likely to report heavy drinking episodes, and further showed an increase in prevalence of heavy episodic drinking during the study period. While the Chinese custom of spirit drinking [12, 13, 32] and drinking with meals remained steady throughout our study period, our findings revealed a rise in drinking mixed beverage types, particularly in younger men. This shift is likely the result of exposure to a wider availability of alcoholic beverages brought by the rapid economic development and westernisation in modern China. Importantly, a rise in drinking multiple beverage types among young Chinese men is of public health concern, since drinkers seemed to drink more in each session when they consumed multiple beverage types.

As in the present study, previous studies in England and Hong Kong also reported the clustering of heavy drinking with smoking, low fruit and vegetables intake and low exercise [33, 34], in settings where the lifestyle behavioural patterns differed from those in mainland of China. Previous studies have shown that people with multiple risk factors, combined with heavy drinking, had higher risks for adverse health outcomes [35–37]. Though the prevalence of smoking may have slightly decreased in China in the last decade, overall cigarette consumption has increased over time [38, 39], as has tobacco-attributed mortality [40]. Given the consistently growing tobacco-attributed burden and alcohol epidemic, particularly among men, and the shift to sedentary lifestyles in China which is accelerated by urbanisation, the consequences of these rapid lifestyle transitions may emerge in an increased chronic disease burden [41].

One important step in alcohol policy development is to understand the triggers for drinking cessation in Chinese populations. Our findings that existing illness was the most reported reason for stopping weekly drinking are generally consistent with a study of 14,000 participants in China in the 1990s [11]. However, concerns about future health were not a common reason to stop drinking in our study, which may indicate a lack of health knowledge about alcohol drinking in China as suggested in a previous study in Chinese elderly men and women [42].

Although not designed to be nationally representative, the diverse geographical regions, large sample size in CKB study and rich data on alcohol drinking patterns covered present a good source of data to assess recent alcohol consumption trends for Chinese adults born in 1920s–1970s. Furthermore, to our knowledge, it is the first study to explore longitudinal changes in alcohol drinking among Chinese adults. However, although the expected associations between alcohol intake and blood pressure observed in this study have provided support for the data quality, potential reporting bias of the self-reported alcohol intake data may still exist. Though older participants were slightly less likely to participate in the resurvey, the resurvey response rates were highly consistent across baseline alcohol drinking groups, suggesting that our findings on temporal trends of alcohol drinking were unlikely to be substantially biased by differential resurvey response rates. However, our results may still be partially influenced by the “sick-quitter” effect, i.e. older drinkers had quit alcohol drinking between the study period and the remaining younger heavy drinkers in the weekly drinker group drove up the mean consumption at resurvey. Furthermore, the measure of alcohol use based on “last time drinking” may have limitations subject to time variation [43–45]. Nevertheless, the use of “last time drinking” as a measure of alcohol intake has been supported by findings from our data quality analyses as well as other studies [44, 46–48].

## Conclusions

In summary, among men who drank regularly, the proportion engaging in harmful drinking behaviours has increased in China over the past decade, particularly among younger men, and heavy intake was associated with having other unhealthy lifestyles and health-related risk factors. As alcohol is a modifiable risk factor with reversible health hazards after stopping drinking in the long term, encouraging heavy drinkers to stop drinking before illness develops could have significant benefits to public health in China over the next decades.

## Additional file

Additional file 1: Supplementary tables and figures. (DOCX 118 kb)

## Abbreviations

CHNS: China Health and Nutrition Survey
CKB: China Kadoorie Biobank
CLHLS: Chinese Longitudinal Healthy Longevity Survey
CNNHS: China National Nutrition and Health Survey
WHO: World Health Organisation
