# Supplementary material for: Patterns and trends of alcohol consumption in rural and urban areas of China: findings from the China Kadoorie Biobank
Source: BMC Public Health. 2019 Feb 20;19:217. doi: 10.1186/s12889-019-6502-1 (PMC6383236; doi:10.1186/s12889-019-6502-1)
Supplement: Supplementary file 1 — Supplementary tables and figures. (DOCX 118 kb) [file 12889_2019_6502_MOESM1_ESM.docx]

**Table S1. Definitions of main alcohol drinking pattern and health-related risk factor variables**

| **Variables** | **Description** |
| --- | --- |
| **Drinking status** | |
| Abstainers^ | Past 12 months: Never drank alcohol.  In the past: Had not drunk weekly. |
| Ex-weekly drinkers | Past 12 months: Never drank alcohol.  In the past: Had drunk weekly. |
| Reduced-intake drinkers | Past 12 months: Had drunk alcohol occasionally, at certain seasons, or monthly but less than weekly.  In the past: Had drunk weekly. |
| Occasional drinkers | Past 12 months: Had drunk alcohol occasionally, at certain seasons, or monthly but less than weekly.  In the past: Had not drunk weekly. |
| Current weekly drinkers | Past 12 months: Weekly (i.e. drank alcohol most weeks).  In the past: --N/A |
| **Drinking patterns** | |
| Drinking frequency | 1-2 days/week; 3-5 days/week; daily or almost every day. |
| Mean consumption^#^ | Calculated based on the beverage type and amount drunk (g of pure alcohol/session).  Alcohol content by volume (v/v) of each beverage type are assumed as the following:[36] beer 4%, grape wine 12%, rice wine 15%, weak spirits 38% and strong spirits 53%.  Note: As the questionnaire data on beverage type and amount consumed (regarding the option of reporting consumption data for multiple alcohol types in resurvey) on the last time drinking (but not on typical drinking day) were directly comparable between the baseline and resurvey, and data reported for a typical drinking day and the last time drinking were highly correlated (**Table S2**), data on the last time the participants drank were used for the main temporal trend analysis in the present study. |
| Types of alcohol^#^ | Strong spirit (≥40% alcohol) only; weak spirit (<40% alcohol) only; wine (rice wine and grape wine) only; beer only; mixed (more than one types, available in resurvey only). |
| Heavy episodic drinking^#^ | Men: consumption of >60 g of alcohol per session;  Women: consumption of >40 g of alcohol per session. |
| Problem drinking indicators | Reporting one or more of the following in the past month: (i) ever drinking in the morning; (ii) unable to work or do anything due to drinking; (iii) depressed, irritated or lost control due to drinking; (iv) couldn’t stop drinking; (v) had shakes when stopped drinking. |
| Flushing response | Experiencing hot flushes or dizziness soon after first mouthful or after drinking small amount of alcohol |
| Drinking with/outside meals | Usually drink with meals; Usually drink between or after meals or no regular patterns |
| **Reasons for stopping weekly drinking** | |
| Reasons | Existing physical illness; money; future health concerns (“future health concerns” and “doctor’s advice” combined); and other (“family against” and “other” combined) |
| **Change in drinking status between baseline and resurvey*** | |
| Stable non-drinkers | Non-drinker at both surveys |
| Starters | Non-drinker at baseline who became drinker at resurvey |
| Stable drinkers | Drinker with the same drinking frequency at both surveys (e.g. all-time occasional or all-time weekly drinkers) |
| Stoppers | Drinker at baseline who became non-drinker at resurvey |
| Decreased-intake drinkers | Drinker at both surveys, but with decreased drinking frequency at resurvey (e.g. changed from weekly to occasional drinkers) |
| Increased-intake drinkers | Drinker at both surveys, but with increased drinking frequency at resurvey (e.g. changed from occasional to weekly drinkers) |
| **Health-related risk factor index** | |
| Risk factor index | Total score summed from four major risk factors (1=yes, 0=no): regular smoking; lack of daily fresh fruit intake; low physical activity (i.e., <17.8 metabolic equivalent tasks hours/day); hypertension (i.e., self-reported or screen-detected with mean systolic blood pressure >140 mmHg or diastolic blood pressure >90 mmHg) |

^Past-year abstainers

*Non-drinkers: abstainers, ex-weekly drinkers; drinkers: reduced-intake drinkers, occasional drinkers, current weekly drinkers.

^#^Data was available on a typical drinking day, on special occasions, and the last time the participants drank. Data on the last time drinking was used in the main temporal trend analysis for methodological consistency.

**Table S2. Cross-tabulation of types of beverage reported on a typical drinking day and on the last drinking day by male weekly drinkers at baseline (2004-2008) and resurvey (2013-2014)**

|  | | | **Types consumed on typical drinking day (%)** | | | | | | | | | | | | | | | | | | | | | | | | | | | | |
| --- | --- | --- | --- | --- | --- | --- | --- | --- | --- | --- | --- | --- | --- | --- | --- | --- | --- | --- | --- | --- | --- | --- | --- | --- | --- | --- | --- | --- | --- | --- | --- |
|  | | | **Baseline** | | | | | | | | | | | |  | | | | **Resurvey** | | | | | | | | | | | | |
| **Types consumed on previous drinking day (%)** |  | | **Beer only** |  | | **Rice or grape wine only** |  | | **Strong spirits** |  | | **Weak spirits only** |  | | | | | | **Beer only** |  | | **Rice or grape wine only** |  | | **Strong spirits** |  | | **Weak spirits only** |  | | **Mixed** |
| Beer only | . | . | 75.3 | . | . | 8.8 | . | . | 6.4 | . | . | 10.0 | . | . | . | . | . | . | 83.0 | . | . | 3.7 | . | . | 3.9 | . | . | 1.6 | . | . | 16.5 |
| Rice or grape wine only | . | . | 2.6 | . | . | 71.9 | . | . | 1.4 | . | . | 5.0 | . | . | . | . | . | . | 3.1 | . | . | 82.3 | . | . | 2.2 | . | . | 3.3 | . | . | 6.3 |
| Strong spirit only | . | . | 7.5 | . | . | 2.1 | . | . | 83.4 | . | . | 3.7 | . | . | . | . | . | . | 6.3 | . | . | 6.7 | . | . | 89.2 | . | . | 1.2 | . | . | 25.4 |
| Weak spirit only | . | . | 4.7 | . | . | 7.9 | . | . | 1.9 | . | . | 74.8 | . | . | . | . | . | . | 0.9 | . | . | 3.7 | . | . | 0.4 | . | . | 91.6 | . | . | 3.9 |
| Mixed | . | . | 9.9 | . | . | 9.3 | . | . | 6.9 | . | . | 6.5 | . | . | . | . | . | . | 6.7 | . | . | 3.7 | . | . | 4.3 | . | . | 2.3 | . | . | 47.9 |

Percentages are unadjusted.

p for chi-square test and Mantel-Haenszel test are <0.001.

In continuous analysis of intake data reported on the previous and typical drinking day, Pearson correlation coefficient is 0.487 (p<0.001) (baseline) and 0.649 (p<0.001) (resurvey).

**Table S3. Association of blood pressure with alcohol consumption among men in the CKB study population at baseline (2004-2008) and resurvey (2013-2014)**

|  | | | | | | | | | | | | | | | | | | | | **Current weekly drinkers by consumption (g) per session** | | | | | | | | | | | | | | | | |
| --- | --- | --- | --- | --- | --- | --- | --- | --- | --- | --- | --- | --- | --- | --- | --- | --- | --- | --- | --- | --- | --- | --- | --- | --- | --- | --- | --- | --- | --- | --- | --- | --- | --- | --- | --- | --- |
|  | | | | **Abstainers** |  | | | **Ex-weekly** |  | | | **Reduced intake** |  | | | **Occasional** |  | | | **<21** |  | | | **21-41** |  | | | **42-62** |  | | | **63+** |  | | | **p-trend^a^** |
| **Mean systolic blood pressure, mmHg (SE)** | | | | | | | | | | | | | | | | | | | | | | | | | | | | | | | | | | | | |
| Baseline | . | . | . | 131.5 (0.10) | . | . | . | 133.2 (0.23) | . | . | . | 133.0 (0.19) | . | . | . | 130.8 (0.07) | . | . | . | 132.1 (0.13) | . | . | . | 134.0 (0.13) | . | . | . | 135.6 (0.16) | . | . | . | 137.4 (0.16) | . | . | . | <0.0001 |
| Resurvey | . | . | . | 134.7 (0.34) | . | . | . | 135.8 (0.85) | . | . | . | 137.1 (1.01) | . | . | . | 134.9 (0.38) | . | . | . | 137.2 (0.70) | . | . | . | 138.7 (0.66) | . | . | . | 139.3 (0.81) | . | . | . | 141.6 (0.73) | . | . | . | <0.0001 |
| **Mean diastolic blood pressure, mmHg (SE)** | | | | | | | | | | | | | | | | | | | | | | | | | | | | | | | | | | | | |
| Baseline | . | . | . | 78.2 (0.06) | . | . | . | 79.4 (0.14) | . | . | . | 79.6 (0.11) | . | . | . | 78.0 (0.04) | . | . | . | 79.0 (0.08) | . | . | . | 80.5 (0.08) | . | . | . | 81.5 (0.10) | . | . | . | 82.9 (0.09) | . | . | . | <0.0001 |
| Resurvey | . | . | . | 78.2 (0.20) | . | . | . | 79.3 (0.50) | . | . | . | 80.1 (0.60) | . | . | . | 78.9 (0.23) | . | . | . | 80.3 (0.41) | . | . | . | 81.2 (0.39) | . | . | . | 81.3 (0.48) | . | . | . | 83.3 (0.43) | . | . | . | <0.0001 |

Means are adjusted for age, regions, education, income, smoking, physical activity and seasonality.

^a^P for trend by consumption category within weekly drinkers.

**Table S4. Baseline characteristics of participants at baseline (2004-8) and resurvey (2013-14)**

|  | **Men** | | |  | | **Women** | | |
| --- | --- | --- | --- | --- | --- | --- | --- | --- |
| **Characteristics** | **CKB study population (N=210259)** |  | **Resurvey subset (N=9569)** |  | | **CKB study population (N=302632)** |  | **Resurvey subset (N=15427)** |
| **Socio-demographic characteristics** | | | | | | | | |
| Mean age, years | 52.4 |  | 51.8 |  |  | 51.0 |  | 50.5 |
| Birth cohorts, % | | | | | | | | |
| <1940 | 13.8 | . | 11.6 | . | . | 10.3 | . | 8.6 |
| 1940-1949 | 22.4 | . | 24.2 | . | . | 20.6 | . | 22.2 |
| 1950-1959 | 30.9 | . | 32.9 | . | . | 32.2 | . | 34.4 |
| 1960-1969 | 27.9 | . | 27.5 | . | . | 31.3 | . | 30.5 |
| ≥1970 | 4.9 | . | 3.9 | . | . | 5.7 | . | 4.3 |
| Area, % | | | | | | | | |
| Rural | 56.6 | . | 56.8 | . | . | 55.4 | . | 57.1 |
| Highest education, % | | | | | | | | |
| No formal education | 8.9 | . | 8.0 | . | . | 25.3 | . | 25.4 |
| Primary school | 33.3 | . | 35.1 | . | . | 31.4 | . | 33.3 |
| Middle or high school | 49.9 | . | 49.3 | . | . | 38.8 | . | 37.4 |
| Technical school/college or above | 7.9 | . | 7.6 | . | . | 4.4 | . | 3.9 |
| Household income (yuan/year), % | | | | | | | | |
| <10,000 | 26.0 | . | 28.3 | . | . | 29.8 | . | 33.7 |
| 10,000-19,999 | 28.3 | . | 30.6 | . | . | 29.6 | . | 31.0 |
| 20,000-34,999 | 25.4 | . | 22.3 | . | . | 24.2 | . | 22.3 |
| 35,000+ | 20.2 | . | 18.8 | . | . | 16.5 | . | 13.0 |
| **Health and lifestyle factors** | | | | | | | | |
| Regular smoking, % | 61.1 | . | 61.4 | . | . | 2.4 | . | 2.1 |
| Daily fruit intake, % | 23.0 | . | 21.8 | . | . | 31.8 | . | 30.4 |
| Physical activity, mean MET hours/day | 22.0 | . | 22.7 | . | . | 20.4 | . | 20.6 |
| Self-reported good health, % | 49.2 | . | 51.2 | . | . | 43.3 | . | 45.2 |
| Prior disease, % | 22.6 | . | 21.1 | . | . | 22.1 | . | 20.6 |
| Satisfied with life, % | 69.7 | . | 68.2 | . | . | 67.7 | . | 65.2 |
| **Physical measurements** | | | | | | | | |
| Mean SBP, mmHg | 132.8 | . | 132.4 | . | . | 129.9 | . | 130.1 |
| Mean DBP, mmHg | 79.2 | . | 78.8 | . | . | 76.8 | . | 76.9 |
| Mean heart rate, beats/minute | 77.7 | . | 77.6 | . | . | 79.7 | . | 79.8 |
| Mean BMI, kg/m^2^ | 23.4 | . | 23.5 | . | . | 23.8 | . | 23.9 |
| Mean WHR | 0.9 | . | 0.9 | . | . | 0.9 | . | 0.9 |
| Mean standing height, cm | 165.2 | . | 165.1 | . | . | 154.1 | . | 154.0 |

BMI, body mass index; S/DBP, systolic/diastolic blood pressure; MET, metabolic equivalent task; WHR, waist-hip ratio.

**Table S5. Response rates at resurvey (2013-2014) by birth cohort, region and baseline drinking group**

|  | **Potentially eligible,  N** | |  | **Invited^^^,  N** | |  | **Enrolled^*^, %** | | |
| --- | --- | --- | --- | --- | --- | --- | --- | --- | --- |
|  |  |  |  |  |  |  | **Men** | **Women** | **Overall** |
| Birth cohorts |  |  |  |  |  |  |  |  |  |
| <1940 | 3690 |  |  | 3585 |  |  | 70 | 66 | 68 |
| 1940-1949 | 7298 |  |  | 7238 |  |  | 80 | 79 | 79 |
| 1950-1959 | 10865 |  |  | 10825 |  |  | 76 | 79 | 78 |
| 1960-1969 | 9999 |  |  | 9985 |  |  | 69 | 76 | 73 |
| 1970+ | 1504 |  |  | 1504 |  |  | 64 | 72 | 69 |
| Region |  |  |  |  |  |  |  |  |  |
| Rural | 17646 |  |  | 17519 |  |  | 78 | 83 | 81 |
| Urban | 15710 |  |  | 15618 |  |  | 68 | 70 | 69 |
| Baseline drinking status |  |  |  |  |  |  |  |  |  |
| Abstainers | 15398 |  |  | 15285 |  |  | 74 | 78 | 77 |
| Ex-weekly | 520 |  |  | 503 |  |  | 76 | 89 | 78 |
| Reduced-intake | 707 |  |  | 700 |  |  | 73 | 83 | 74 |
| Occasional | 11750 |  |  | 11696 |  |  | 73 | 74 | 74 |
| Weekly | 4981 |  |  | 4953 |  |  | 73 | 76 | 74 |
| Overall | 33356 |  |  | 33137 |  |  | 73 | 77 | 75 |

^Of all potentially eligible participants, 0.7% (0.9% men, 0.5% women) had died by the time of resurvey and thus were not invited to participate.

*Proportion of the invited participants who had enrolled and had completed questionnaire data.

**Table S6. Prevalence of drinking with meals among male weekly drinkers between baseline (2004-2008) and resurvey (2013-2014), by region**

|  | | | **Baseline** | | |  | |  | | **Resurvey** | | | | |
| --- | --- | --- | --- | --- | --- | --- | --- | --- | --- | --- | --- | --- | --- | --- |
|  | | | **N** |  | **Drinking with meals %** | |  | |  | | **N** |  | **Drinking with meals %** |  |
| Qingdao (Urban) | . | . | 7501 | . | 80.2 | | . | | . | | 230 | . | 92.2 | . |
| Harbin (Urban) | . | . | 11506 | . | 87.4 | | . | | . | | 323 | . | 89.1 | . |
| Haikou (Urban) | . | . | 1703 | . | 84.2 | | . | | . | | 134 | . | 82.8 | . |
| Suzhou (Urban) | . | . | 9103 | . | 98.6 | | . | | . | | 421 | . | 81.2 | . |
| Liuzhou (Urban) | . | . | 5210 | . | 90.5 | | . | | . | | 216 | . | 74.0 | . |
| Sichuan (Rural) | . | . | 10739 | . | 98.9 | | . | | . | | 457 | . | 98.6 | . |
| Gansu (Rural) | . | . | 1475 | . | 19.3 | | . | | . | | 85 | . | 21.1 | . |
| Henan (Rural) | . | . | 7092 | . | 69.3 | | . | | . | | 374 | . | 69.9 | . |
| Zhejiang (Rural) | . | . | 9174 | . | 98.4 | | . | | . | | 288 | . | 97.2 | . |
| Hunan (Rural) | . | . | 6401 | . | 63.2 | | . | | . | | 204 | . | 61.1 | . |

Prevalences are adjusted for age.

**Table S7. Changes in alcohol consumption (g per session) between baseline and resurvey at the individual level among men who were weekly drinkers at both time points, by socio-demographic characteristics**

|  | **N** | **Resurvey – Baseline**  **Mean difference**  **g/session** | **p-value** |
| --- | --- | --- | --- |
| Overall | 2042 | 3.7 (1.2 - 6.1) | 0.004 |
| Birth cohorts |  |  |  |
| <1940 | 148 | 1.3 (-4.2 - 6.9) | 0.632 |
| 1940-1949 | 420 | 1.3 (-3.0 - 5.7) | 0.542 |
| 1950-1959 | 784 | 2.3 (-1.5 - 6.1) | 0.238 |
| 1960-1969 | 610 | 5.7 (0.3 - 11.0) | 0.040 |
| ≥1970 | 80 | 18.2 (2.3 - 34.2) | 0.026 |
| Region |  |  |  |
| Rural | 1034 | 1.8 (-1.0 - 4.6) | 0.210 |
| Urban | 1008 | 5.6 (1.5 - 9.6) | 0.007 |
| Highest education |  |  |  |
| Primary or below | 854 | 3.5 (0.0 – 7.0) | 0.048 |
| Middle or above | 1188 | 3.8 (0.3 – 7.2) | 0.031 |
| Household income (yuan/year) | | | |
| <35,000 | 665 | 0.0 (-3.7 - 3.7) | 0.996 |
| 35,000+ | 1377 | 5.4 (2.2 - 8.6) | 0.001 |

Significance for change in alcohol consumption over time was tested using dependent sample t-test.

**Table S8. Mean consumption in grams per day or session by male weekly drinkers of different beverage reporting categories at baseline (2004-2008) and resurvey (2013-2014)**

|  | | **Baseline** | | | | | | | | | | | | | |  | **Resurvey** | | | | | | | | | | | | | |
| --- | --- | --- | --- | --- | --- | --- | --- | --- | --- | --- | --- | --- | --- | --- | --- | --- | --- | --- | --- | --- | --- | --- | --- | --- | --- | --- | --- | --- | --- | --- |
|  | | **Typical day** | | | |  | **Previous drinking day** | | | |  | **Special occasion** | | | |  | **Typical day** | | | |  | **Previous drinking day** | | | |  | **Special occasion** | | | |
|  | | **N** |  | **Mean, g/day (SD)** | |  | **N** |  | **Mean, g/session (SD)** | |  | **N** |  | **Mean, g/session (SD)** | |  | **N** |  | **Mean, g/day (SD)** | |  | **N** |  | **Mean, g/session (SD)** | |  | **N** |  | **Mean, g/session (SD)** | |
| Overall | . | 69904 | . | 54.3 | (0.14) | . | 69904 | . | 49.9 | (0.15) | . | 69904 | . | 147.4 | (0.41) | . | 2732 | . | 65.6 | (1.01) | . | 2732 | . | 55.6 | (0.92) | . | 2732 | . | 116.3 | (1.76) |
| **Mixed drinkers** | | | | | | | | | | | | | | | | | | | | | | | | | | | | | | |
| Non-mixed | . | 69904 | . | 54.3 | (0.14) | . | 64544 | . | 45.6 | (33.91) | . | 48584 | . | 125.6 | (87.65) | . | 1968 | . | 52.1 | (37.84) | . | 2286 | . | 46.4 | (34.71) | . | 1864 | . | 90.7 | (66.63) |
| Mixed | . | --* | . | --* |  | . | 5360 | . | 101.0 | (59.12) | . | 21320 | . | 196.9 | (133.98) | . | 764 | . | 100.3 | (67.36) | . | 446 | . | 103.0 | (72.89) | . | 868 | . | 171.3 | (113.19) |
| **One-type drinkers** | | | | | | | | | | | | | | | | | | | | | | | | | | | | | | |
| Beer only | . | 12725 | . | 38.2 | (25.55) | . | 14025 | . | 39.1 | (32.33) | . | 7617 | . | 100.9 | (65.62) | . | 223 | . | 37.8 | (28.08) | . | 369 | . | 42.0 | (40.70) | . | 230 | . | 79.3 | (58.46) |
| Rice wine or grape wine only | . | 8505 | . | 35.0 | (26.59) | . | 7695 | . | 28.3 | (21.67) | . | 4634 | . | 59.3 | (47.91) | . | 299 | . | 31.7 | (27.65) | . | 337 | . | 28.3 | (23.81) | . | 254 | . | 44.1 | (39.96) |
| Strong spirit only | . | 32727 | . | 64.9 | (40.11) | . | 29003 | . | 53.1 | (36.77) | . | 24924 | . | 151.4 | (96.98) | . | 1019 | . | 61.4 | (40.33) | . | 1142 | . | 52.8 | (34.40) | . | 979 | . | 110.2 | (72.35) |
| Weak spirit only | . | 15947 | . | 55.8 | (34.33) | . | 13821 | . | 46.1 | (29.72) | . | 11409 | . | 112.8 | (67.43) | . | 427 | . | 51.6 | (34.23) | . | 438 | . | 46.9 | (31.67) | . | 401 | . | 78.9 | (48.82) |

SD, standard deviation.

Means are unadjusted.

*Data not available.

**Table S9. Prevalence and patterns of alcohol consumption at baseline (2004-2008) and resurvey (2013-2014) among the subset of participants who participated in both surveys, by sex**

|  | | **Men** | |  | **Women** | |
| --- | --- | --- | --- | --- | --- | --- |
|  | | **Baseline** | **Resurvey** |  | **Baseline** | **Resurvey** |
| **Overall** | | | | | | |
| Number of participants | . | 9569 | 9569 | . | 15427 | 15427 |
| Drinking categories, % | | | | | | |
| Abstainer | . | 19.3 | 34.9 | . | 64.6 | 82.0 |
| Ex-weekly | . | 3.3 | 6.3 | . | 0.5 | 0.7 |
| Reduced intake | . | 4.7 | 3.8 | . | 0.4 | 0.5 |
| Occasional | . | 38.2 | 26.4 | . | 32.2 | 14.9 |
| Current weekly | . | 34.5 | 28.6 | . | 2.2 | 1.9 |
| **Among current weekly drinkers** | | | | | | |
| Number of participants | . | 3301 | 2732 | . | 347 | 292 |
| Types consumed , % | | | | | | |
| Strong spirit (≥40% alcohol) only | . | 39.4 | 41.8 | . | 57.1 | 39.4 |
| Weak spirit (<40% alcohol) only | . | 20.7 | 16.0 | . | 7.8 | 12.0 |
| Beer only | . | 19.0 | 13.5 | . | 18.4 | 12.3 |
| Rice wine or grape wine only | . | 11.3 | 12.3 | . | 15.0 | 27.1 |
| Mixed | . | 9.7 | 16.3 | . | 1.7 | 9.2 |
| Mean consumption, g/session | . | 51.8 | 55.6 | . | 21.5 | 24.1 |
| Number of drinking days per week, % | | | | | | |
| 1-2 | . | 20.6 | 14.9 | . | 26.5 | 21.9 |
| 3-5 | . | 19.0 | 14.0 | . | 20.5 | 15.4 |
| 6-7 | . | 60.4 | 71.1 | . | 53.0 | 62.7 |
| Types consumed on special occasions, % | | | | | | |
| Strong spirit (≥40% alcohol) only | . | 34.0 | 35.8 | . | 51.0 | 33.9 |
| Weak spirit (<40% alcohol) only | . | 16.2 | 14.7 | . | 6.3 | 10.6 |
| Beer only | . | 9.2 | 8.4 | . | 9.2 | 11.0 |
| Rice wine or grape wine only | . | 6.1 | 9.3 | . | 13.0 | 25.7 |
| Mixed | . | 34.5 | 31.8 | . | 20.5 | 18.8 |
| Mean consumption on special occasions, g/occasion | . | 147.6 | 116.3 | . | 55.2 | 41.3 |
| Drinking patterns, % | | | | | | |
| Drinking outside meal | . | 15.9 | 17.6 | . | 13.5 | 20.9 |
| Heavy episodic drinking | . | 31.0 | 34.7 | . | 15.0 | 17.8 |
| Heavy episodic drinking on special occasions | . | 84.5 | 72.9 | . | 57.1 | 43.8 |
| Problem drinking indicator(s) | . | 26.0 | 23.8 | . | 13.8 | 9.2 |
| Flushing response after drinking | . | 19.7 | 15.5 | . | 23.3 | 13.7 |
| Mean age started weekly drinking | . | 28.8 | 29.3 | . | 37.5 | 40.4 |

**Figure S1. Alcohol drinking characteristics in male weekly drinkers in 2004-8 and 2013-4, by age group**

Percentages are adjusted for regions.

**Table S10. Demographic and socio-economic correlates of drinking between baseline (2004-2008) and resurvey (2013-2014), by sex**

|  | | **Baseline** | | | | | | | | |  | | | **Resurvey** | | | | | | | | | | | | | |  |
| --- | --- | --- | --- | --- | --- | --- | --- | --- | --- | --- | --- | --- | --- | --- | --- | --- | --- | --- | --- | --- | --- | --- | --- | --- | --- | --- | --- | --- |
|  | | | | | | | **Weekly** | | | |  | | | | | | | | | | | | | **Weekly** | | | |  |
|  | | **N** | **Abstainer %** | **Ex-weekly %** | **Reduced intake %** | **Occasional %** | **%** | | **Mean consumption**^*^**(SE)** | |  | | | **N** | **Abstainer %** | | **Ex-weekly %** | | **Reduced intake %** | | | **Occasional %** | | **%** | **Mean consumption**^*^**(SE)** | | |  |
| **Men** | | | | | | | | | | | | | | | | | | | | | | | | | | | |  |
| Birth cohorts | | | | | | | | | | | | | | | | | | | | | | | | | | | |  |
| <1940 | . | 29036 | 32.3 | 7.8 | 5.7 | 29.8 | 24.4 | | 34 | (0.3) | . | | | 1106 | 53.1 | | 10.4 | | 2.7 | | | 15.4 | | 18.4 | 36 | | (2.1) |  |
| 1940-1949 | . | 47200 | 24.4 | 5.7 | 6.5 | 33.5 | 30.0 | | 42 | (0.3) | . | | | 2311 | 41.0 | | 10.4 | | 4.7 | | | 19.9 | | 24.0 | 45 | | (1.8) |  |
| 1950-1959 | . | 64939 | 18.1 | 3.0 | 5.2 | 37.3 | 36.4 | | 51 | (0.3) | . | | | 3149 | 32.8 | | 5.6 | | 4.4 | | | 25.9 | | 31.3 | 54 | | (1.4) |  |
| 1960-1969 | . | 58690 | 14.6 | 1.5 | 3.5 | 43.7 | 36.7 | | 57 | (0.3) | . | | | 2632 | 26.8 | | 2.6 | | 2.8 | | | 35.7 | | 32.2 | 66 | | (1.9) |  |
| ≥1970 | . | 10394 | 17.3 | 0.7 | 2.7 | 52.3 | 27.0 | | 58 | (0.8) | . | | | 371 | 23.1 | | 1.2 | | 4.2 | | | 40.1 | | 31.5 | 77 | | (7.0) |  |
| Area | | | | | | | | | | | | | | | | | | | | | | | | | | | |  |
| Rural | . | 118903 | 24.2 | 4.3 | 4.6 | 37.5 | 29.3 | | 49 | (0.2) | . | | | 5433 | 39.2 | | 7.2 | | 3.3 | | | 24.5 | | 25.9 | 51 | | (1.0) |  |
| Urban | . | 91356 | 15.3 | 3.1 | 5.3 | 37.9 | 38.3 | | 51 | (0.2) | . | | | 4136 | 29.4 | | 5.2 | | 4.5 | | | 28.9 | | 32.0 | 60 | | (1.5) |  |
| Highest education | | | | | | | | | | | | | | | | | | | | | | | | | | | |  |
| Primary or below | . | 88788 | 22.8 | 4.5 | 4.9 | 34.6 | 33.2 | | 49 | (0.7) | . | | | 4076 | 37.6 | | 6.6 | | 3.2 | | | 23.9 | | 28.7 | 56 | | (2.0) |  |
| Middle or above | . | 121471 | 19.0 | 3.2 | 5.2 | 39.9 | 32.6 | | 50 | (0.2) | . | | | 5493 | 33.5 | | 5.7 | | 4.1 | | | 28.2 | | 28.4 | 54 | | (1.2) |  |
| Household income (yuan/year) | | | | | | | | | | | | | | | | | | | | | | | | | | | |  |
| <20,000 | . | 114313 | 21.9 | 4.3 | 4.6 | 36.8 | 32.3 | | 49 | (0.3) | . | | | 1858 | 39.5 | | 7.6 | | 3.9 | | | 22.9 | | 26.0 | 48 | | (2.4) |  |
| 20,000+ | . | 95946 | 18.5 | 3.3 | 5.5 | 37.9 | 34.8 | | 52 | (0.3) | . | | | 7711 | 34.3 | | 5.8 | | 3.9 | | | 26.9 | | 29.0 | 56 | | (1.0) |  |
| **Women** | | | | | | | | | | | | | | | | | | | | | | | | | | | | |
| Birth cohorts | | | | | | | | | | | | | | | | | | | | | | | | | | | | |
| <1940 | . | 31109 | 72.9 | 1.1 | 0.6 | 22.7 | 2.7 | 16 | | (3.9) | | . | 1320 | | | 87.9 | | 1.6 | | 0.4 | 7.2 | | 3.0 | | | 18 | (15.5) | |
| 1940-1949 | . | 62246 | 69.1 | 0.8 | 0.6 | 27.4 | 2.2 | 17 | | (2.7) | | . | 3431 | | | 86.5 | | 1.3 | | 0.5 | 9.9 | | 1.8 | | | 26 | (16.4) | |
| 1950-1959 | . | 97344 | 63.0 | 0.4 | 0.5 | 33.9 | 2.2 | 23 | | (2.8) | | . | 5303 | | | 83.0 | | 0.7 | | 0.7 | 13.8 | | 1.9 | | | 22 | (12.1) | |
| 1960-1969 | . | 94772 | 58.0 | 0.1 | 0.3 | 39.8 | 1.8 | 27 | | (3.0) | | . | 4702 | | | 76.1 | | 0.4 | | 0.4 | 21.5 | | 1.7 | | | 24 | (15.6) | |
| ≥1970 | . | 17161 | 54.3 | 0.0 | 0.1 | 43.0 | 2.6 | 25 | | (9.3) | | . | 671 | | | 71.3 | | 0.1 | | 0.5 | 26.1 | | 2.1 | | | 35 | (7.8) | |
| Area | | | | | | | | | | | | | | | | | | | | | | | | | | | | |
| Rural | . | 167802 | 65.4 | 0.7 | 0.6 | 31.3 | 2.1 | 23 | | (2.4) | | . | 8802 | | | 85.5 | | 1.0 | | 0.4 | 11.1 | | 2.0 | | | 25 | (10.0) | |
| Urban | . | 134830 | 60.9 | 0.2 | 0.3 | 36.4 | 2.1 | 21 | | (1.5) | | . | 6625 | | | 76.7 | | 0.5 | | 0.7 | 20.3 | | 1.8 | | | 24 | (11.5) | |
| Highest education | | | | | | | | | | | | | | | | | | | | | | | | | | | | |
| Primary or below | . | 171649 | 68.8 | 0.5 | 0.5 | 28.4 | 1.9 | 25 | | (4.5) | | . | 9016 | | | 84.1 | | 0.7 | | 0.5 | 13.0 | | 1.7 | | | 22 | (7.8) | |
| Middle or above | . | 130983 | 59.7 | 0.4 | 0.5 | 37.4 | 2.1 | 22 | | (3.4) | | . | 6411 | | | 79.5 | | 0.5 | | 0.7 | 17.3 | | 2.0 | | | 21 | (11.6) | |
| Household income (yuan/year) | | | | | | | | | | | | | | | | | | | | | | | | | | | | |
| <20,000 | . | 179532 | 65.9 | 0.4 | 0.4 | 31.4 | 1.9 | 22 | | (2.3) | | . | 3390 | | | 84.3 | | 0.5 | | 1.0 | 12.4 | | 1.8 | | | 19 | (10.8) | |
| 20,000+ | . | 123100 | 59.7 | 0.4 | 0.5 | 37.2 | 2.2 | 23 | | (4.9) | | . | 12037 | | | 81.4 | | 0.8 | | 0.4 | 15.5 | | 1.8 | | | 22 | (8.4) | |

SE, standard error.

Prevalences and means are adjusted for age and regions.

^*^Pure alcohol in grams/session.

**Table S11. Drinking patterns among male weekly drinkers at baseline (2004-2008) and resurvey (2013-2014), by socio-demographic characteristics**

|  | | | **Baseline** | | | | | | |  | **Resurvey** | | | | | | |
| --- | --- | --- | --- | --- | --- | --- | --- | --- | --- | --- | --- | --- | --- | --- | --- | --- | --- |
|  | | | **N** |  | **Daily drinking %** | **Drinking outside meals %** | **Heavy episodic drinking %** | **Heavy episodic drinking on special occasions %** | **Problem drinking indicator(s) %** |  | **N** |  | **Daily drinking %** | **Drinking outside meals %** | **Heavy episodic drinking  %** | **Heavy episodic drinking on special occasions %** | **Problem drinking indicator(s) %** |
| **Socio-demographic characteristics** | | | | | | | | | | | | | | | | | |
| Birth cohorts | | | | | | | | | | | | | | | | | |
| <1940 | . | . | 7109 | . | 77.3 | 15.3 | 12.4 | 65.6 | 23.5 | . | 204 | . | 87.5 | 13.2 | 15.0 | 45.9 | 12.6 |
| 1940-1949 | . | . | 14047 | . | 72.8 | 14.0 | 20.5 | 77.5 | 25.2 | . | 565 | . | 78.9 | 16.1 | 23.9 | 62.2 | 23.5 |
| 1950-1959 | . | . | 23987 | . | 64.9 | 14.3 | 31.3 | 86.2 | 25.3 | . | 999 | . | 75.0 | 20.0 | 34.3 | 75.4 | 26.0 |
| 1960-1969 | . | . | 21622 | . | 50.1 | 13.6 | 37.5 | 89.6 | 21.7 | . | 845 | . | 59.3 | 16.9 | 44.1 | 82.3 | 23.7 |
| ≥1970 | . | . | 3139 | . | 36.3 | 14.8 | 40.8 | 93.4 | 17.5 | . | 119 | . | 45.0 | 18.8 | 47.3 | 88.5 | 22.0 |
| Area | | | | | | | | | | | | | | | | | |
| Rural | . | . | 34881 | . | 64.2 | 17.3 | 28.6 | 85.7 | 33.9 | . | 1408 | . | 72.6 | 19.1 | 33.3 | 79.0 | 26.3 |
| Urban | . | . | 35023 | . | 60.1 | 10.9 | 30.9 | 81.1 | 13.9 | . | 1324 | . | 69.0 | 15.9 | 36.2 | 66.5 | 21.3 |
| Highest education | | | | | | | | | | | | | | | | | |
| Primary or below | . | . | 27770 | . | 69.5 | 14.6 | 29.2 | 81.0 | 28.1 | . | 1118 | . | 75.2 | 19.2 | 33.9 | 71.3 | 25.2 |
| Middle or above | . | . | 42134 | . | 59.5 | 13.8 | 29.9 | 84.0 | 21.7 | . | 1614 | . | 70.0 | 17.3 | 33.4 | 75.1 | 21.1 |
| Household income (yuan/year) | | | | | | | | | | | | | | | | | |
| <20,000 | . | . | 33899 | . | 64.9 | 14.7 | 28.9 | 81.5 | 25.9 | . | 462 | . | 74.2 | 17.2 | 27.8 | 70.0 | 22.0 |
| 20,000+ | . | . | 36005 | . | 59.4 | 13.2 | 32.5 | 85.4 | 21.3 | . | 2270 | . | 71.1 | 17.7 | 35.2 | 73.1 | 23.7 |

Prevalences are adjusted for age and regions.

**Table S12. Prevalence of reporting problem drinking indicators among male weekly drinkers at baseline (2004-2008) and resurvey (2013-2014), by birth cohort and region**

|  |  | **Baseline** | | |  | **Resurvey** | | |
| --- | --- | --- | --- | --- | --- | --- | --- | --- |
|  |  | **N** | **Problem drinking indicator(s)**  **%** | |  | **N** | **Problem drinking indicator(s)**  **%** | |
| **Rural** |  |  |  |  |  |  |  |  |
| <1950 |  | 11300 | 36.1 |  |  | 396 | 25.0 |  |
| 1950-1959 |  | 11528 | 35.9 |  |  | 470 | 29.6 |  |
| ≥1960 |  | 12053 | 30.1 |  |  | 542 | 24.0 |  |
|  |  |  |  |  |  |  |  |  |
| **Urban** |  |  |  |  |  |  |  |  |
| <1950 |  | 9856 | 15.2 |  |  | 373 | 17.2 |  |
| 1950-1959 |  | 12459 | 14.3 |  |  | 529 | 22.3 |  |
| ≥1960 |  | 12708 | 12.4 |  |  | 422 | 23.5 |  |

Prevalences are unadjusted.

**Figure S2. Proportions of types of alcohol consumed in male weekly drinkers at baseline (2004-2008) and resurvey (2013-2014), by birth cohort**

**Resurvey**

**Baseline**

Percentages are adjusted for regions.

**Figure S3. Alcohol drinking characteristics in male weekly drinkers in 2004-8 and 2013-4, by health-related risk factor index (hypertension removed)**

Percentages are adjusted for regions.

**Table S13. Changes in drinking by socio-demographic characteristics and health factors among men from baseline to resurvey, based on baseline characteristics**

|  | | | **Non-drinkers (abstainers, ex-weekly drinkers) at baseline^^^** | |  | **Drinkers (reduced-intake, occasional and weekly drinkers) at baseline^#^** | | | |
| --- | --- | --- | --- | --- | --- | --- | --- | --- | --- |
| **Characteristics** |  | **N** | **Stable non-drinker %** | **Starter %** |  | **Stable drinker %** | **Stopper %** | **Decreased-intake drinker %** | **Increased-intake drinker %** |
| All men | . | 9569 | 18.3 | 4.2 | . | 40.0 | 22.9 | 8.2 | 6.3 |
| **Socio-demographic characteristics** | | | | | | | | | |
| Birth cohorts | | | | | | | | | |
| <1940 | . | 1106 | 33.0 | 4.3 | . | 24.0 | 30.6 | 3.9 | 4.3 |
| 1940-1949 | . | 2311 | 23.1 | 4.3 | . | 32.4 | 28.3 | 6.9 | 5.2 |
| 1950-1959 | . | 3149 | 16.3 | 3.8 | . | 43.7 | 22.1 | 8.2 | 6.0 |
| 1960-1969 | . | 2632 | 11.3 | 4.6 | . | 47.3 | 18.1 | 10.7 | 8.0 |
| ≥1970 | . | 371 | 8.3 | 3.7 | . | 50.3 | 16.0 | 11.7 | 10.0 |
| Area | | | | | | | | | |
| Rural | . | 5433 | 21.9 | 4.3 | . | 36.3 | 24.5 | 6.9 | 6.1 |
| Urban | . | 4136 | 13.6 | 4.1 | . | 44.8 | 20.9 | 10.0 | 6.6 |
| Highest education level | | | | | | | | | |
| Primary or below | . | 4121 | 20.4 | 4.0 | . | 38.9 | 23.7 | 6.8 | 6.3 |
| Middle or above | . | 5448 | 16.6 | 4.0 | . | 42.1 | 22.6 | 8.4 | 6.3 |
| Income level (yuan/year) | | | | | | | | | |
| <20,000 | . | 5639 | 19.1 | 4.5 | . | 39.2 | 22.9 | 7.4 | 6.8 |
| 20,000+ | . | 3930 | 17.0 | 3.2 | . | 43.2 | 19.5 | 10.6 | 6.5 |
| **Health and lifestyle factors** | | | | | | | | | |
| Self-reported health | | | | | | | | | |
| Good | . | 4898 | 15.9 | 3.3 | . | 42.8 | 22.4 | 9.2 | 6.3 |
| Poor | . | 4671 | 21.1 | 5.0 | . | 36.6 | 23.5 | 7.5 | 6.3 |
| Prior disease | | | | | | | | | |
| No | . | 7550 | 17.0 | 4.0 | . | 41.2 | 22.7 | 8.8 | 6.3 |
| Yes | . | 2019 | 23.0 | 5.6 | . | 36.1 | 23.1 | 6.2 | 6.0 |
| Risk factor index score | | | | | | | | | |
| 0 | . | 3911 | 17.7 | 4.5 | . | 39.3 | 23.6 | 8.5 | 6.3 |
| 1 | . | 3619 | 17.7 | 3.4 | . | 42.4 | 21.9 | 8.7 | 6.0 |
| 2 | . | 285 | 16.3 | 4.0 | . | 36.2 | 25.3 | 6.1 | 11.9 |
| 3+ | . | 1754 | 20.1 | 5.1 | . | 38.3 | 22.2 | 7.7 | 6.5 |

Prevalence at subgroup levels is adjusted for age and regions.

^^^Among baseline non-drinkers, associations between change in drinking status and factors were tested by logistic regression adjusting for age and regions: p<0.001 for significant trend across birth cohorts and heterogeneity across regions.

^#^Among baseline drinkers, associations between change in drinking status and factors were tested by multinomial logistic regression adjusting for age and regions: p<0.02 across all variables except education.
